# Multiple time‐dependent pathophysiological changes in a rabbit model of high‐fat diet‐induced hyperlipidemia

**DOI:** 10.1002/2211-5463.13597

**Published:** 2023-04-20

**Authors:** Gwang‐Hoon Lee, Kyung‐Ku Kang, Hyun Ho Yun, Woori Jo, Taeho Oh

**Affiliations:** ^1^ Preclinical Research Center Daegu‐Gyeongbuk Medical Innovation Foundation Korea; ^2^ Department of Veterinary Internal Medicine, College of Veterinary Medicine Kyungpook National University Daegu Korea

**Keywords:** atherosclerosis, cardiac contractility, high‐density lipoprotein, high‐fat diet, hyperlipidemia, renal dysfunction

## Abstract

High‐fat diets (HFD) adversely affect organ systems. Several studies have examined HFD‐related disorders in animals but only in a few organs and time points. Herein, we evaluated disease development with time‐dependent HFD‐induced pathological, cardiovascular, and morphological changes in rabbits with lipid metabolism similar to that in humans for 9 weeks. The body weights and waist ratio of the HFD group were higher than those in the control group. HFD significantly increased the total cholesterol, low‐density lipoprotein, high‐density lipoprotein, and phospholipid levels after 3 weeks. Liver enzyme levels increased with hepatomegaly, steatosis, and fibrosis after 3 or 6 weeks. RBCs and hemoglobin decreased, while platelets increased in the HFD group with atherosclerosis and inflammatory cell infiltration in the aorta after 6 weeks. Ejection fraction and fractional shortening values decreased in the HFD group after 9 weeks. Creatinine increased with glomerulosclerosis in the kidneys of the HFD groups after 3 weeks, indicating renal dysfunction. Lipid accumulation was found in the pancreas after 9 weeks. Lipid accumulation and hypertrophy were observed in the adrenal glands after 3 weeks. Overall, our findings provide global reference data on the time‐dependent effects of HFD on the body and may serve as a guide for future HFD risk prevention.

## Graphical abstract

High‐fat diet (HFD) increased body weight and waist ratio. HFD induced hyperlipidemia and an increase in liver enzymes with hepatomegaly, steatosis, and fibrosis. HFD induced a decrease in RBCs and hemoglobin, and an increase in platelets with atherosclerosis in the aorta. HFD induced a decrease in cardiac contractility and an increase in creatinine with glomerulosclerosis. HFD induced lipid accumulation in the pancreas and adrenal glands.

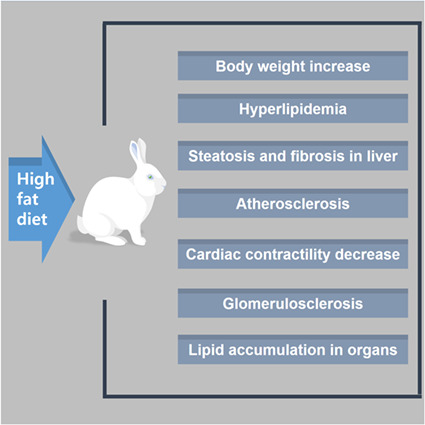

**Abbreviations**
ALT: alanine aminotransferase
AST: aspartate aminotransferase
BUN: blood urea nitrogen
CETP: cholesteryl ester transfer protein
EF: ejection fraction
ESC/EAS: European Society of Cardiology/European Atherosclerosis Society
FS: fractional shortening
H&E: hematoxylin and eosin
HDL: high‐density lipoprotein
HDLs: high‐density lipoproteins
HFD: high‐fat diets
IACUC: Institutional Animal Care and Use Committee
LDL: low‐density lipoprotein
LDLs: low‐density lipoproteins
MAP: mean arterial pressure
NAFLD: nonalcoholic fatty liver disease
NEFA: high‐plasma nonesterified fatty acid
NZW: New Zealand White
RBC: red blood cells
ROS: reactive oxygen species
SEM: standard error of the mean
SPF: specific pathogen‐free
TCHOL: total cholesterol
TG: triglyceride
vWF: von Willebrand factor

High‐fat diet (HFD)‐induced obesity has become a global epidemic and is a risk factor for metabolic disorders, such as cardiovascular disease, neurovascular disease, diabetes mellitus dyslipidemia, metabolic syndrome, and fatty liver disease [1, 2, 3, 4, 5, 6]. The prevalence of Class III obesity has steadily increased in the past 40 years, reaching epidemic proportions worldwide. More than 1.9 billion adults over the age of 18 are overweight (39%), and 650 million adults are obese (13%) [7]. For every 1% increase in obesity prevalence, the mortality rate for people infected with COVID‐19 increased by 8.3% and the incidence by 6.6% [8]. Obesity can be caused by a modern lifestyle dominated by sedentary activities and increasing food overconsumption in the absence of hunger [9]. Owing to the large increase in the worldwide prevalence of obesity, it is important to elucidate the comprehensive adverse effects of an HFD [1, 10, 11].

Characterizing animal disease models is a significant technique for advancing clinical intervention development and understanding disease pathophysiology [1, 12]. Considering the characteristics of each laboratory animal species, the most appropriate animal species should be studied to stimulate human diseases with similar pathophysiologies and complications and discover innovative prevention, treatment, and monitoring measures. Rodents are the predominant animal models for preclinical studies owing to their short lifespan, high fertility, and short duration for disease progression [13, 14, 15, 16]. However, rodents are not suitable laboratory animals for studying HFD‐induced biological changes. Rodents lack plasma cholesteryl ester transfer protein (CETP), which regulates lipoprotein metabolism, and possess more high‐density lipoproteins (HDLs) than low‐density lipoproteins (LDLs), unlike humans [1, 17, 18]. By contrast, rabbits are a well‐known animal model for studying HFD‐induced dyslipidemia or atherosclerosis as their lipoprotein metabolism is similar to that of humans. Rabbits have abundant plasma CETP and LDLs‐rich lipoprotein profiles and high plasma nonesterified fatty acid (NEFA) and triglyceride (TG) levels, similar to those observed in humans with obesity [1, 17]. In addition, rabbits are suitable laboratory animals for studying changes in heart function because the heartbeat mechanism in rabbits is more similar to that in humans than in rodents. The action potential morphology, repolarization mechanism mediated by rectifier K+ currents, and Ca^2+^ transporter function in rabbit ventricular myocardium are more similar to those in the human ventricle than in the rodent ventricle. Therefore, rabbits are widely used to study heart diseases, such as aortic constriction, myocardial ischemia followed by reperfusion, and atrial fibrillation [19, 20, 21, 22].

Although some studies have released data on HDF‐induced changes in rabbits, their findings are fragmented. For example, Abdelhalim and Moussa studied only biochemical changes in red blood cells (RBC) after HFD, Zarzoso *et al*. revealed only HFD‐induced ventricular changes, and Birkner *et al*. studied only the liver for metabolic and antioxidative changes [4, 23, 24]. In addition, observing changes in two or more factors cannot validate the change based on the cumulative exposure time of HFD. Sibouakaz *et al*. analyzed the results of biochemical assays, thin‐layer chromatography, and transmission electron microscopy. However, time‐course changes in the body after HFD exposure were not confirmed because these analyses were performed only 3 months after HFD induction. Shao *et al*. observed growth, behavior, and serum biochemical and morphological changes after an HFD. Although many factors were observed, only the changes after 5 weeks of HFD were confirmed [25, 26].

In this study, a time‐dependent rabbit hyperlipidemic model was designed to investigate comprehensive, time‐dependent pathophysiological changes, including blood components, cardiovascular system, and histopathologic evaluation, for 9 weeks. This early‐phase hyperlipidemic rabbit model will increase our understanding of the progression of this disease in the body after HFD and aid the development of drugs for patients with metabolic syndrome in the early stages of hyperlipidemia. Furthermore, therapeutic strategies for the prevention of metabolic syndrome are expected to improve clinical outcomes in patients.

## Materials and methods

### Husbandry

Male specific‐pathogen‐free (SPF) New Zealand White (NZW) rabbits with an average weight of 2.5 kg were used in this study. The NZW rabbits were bred in a room with 50 ± 20% humidity, 21 ± 3 °C temperature, 10–20 cycles·h^−1^ ventilation rate, and a 12‐h light/dark cycle. After 1 week of acclimatization, the rabbits were randomly divided into HFD and control groups. The control group (*n* = 11) was fed normal rabbit chow (#38302, Purina Rabbit Chow, Purina Co., Ltd., St. Louis, MO, USA), and the animals were divided into three subgroups based on the diet period [3 weeks (*n* = 3), 6 weeks (*n* = 4), and 9 weeks (*n* = 4)]. The rabbits in the HFD group (*n* = 22) were fed 250 g of pellets of a custom‐made Purina Rabbit Chow (#5321, supplemented with 1% cholesterol and 5% corn oil) every day and divided into three subgroups based on the diet period [3 weeks (*n* = 6), 6 weeks (*n* = 8), and 9 weeks (*n* = 7)]. All rabbits were provided with water *ad libitum*. The rabbits were euthanatized on the last day of their dietary schedule for histopathological analysis and autopsy.

This study was approved by the K‐MEDI Hub Institutional Animal Care and Use Committee (IACUC approval number: DGMIF‐17061001, approval date: September 13, 2017). All animal experiments were conducted per the Regulations for Animal Experimentation of the K‐MEDI hub.

### Body weight and waist ratio

The body weights of all animals were determined at 0 (prediet), 1, 2, 3, 6, and 9 weeks during the experimental period. To assess waist ratio changes, X‐ray digital radiography (ELMO‐T3, DK Medical Systems, Seoul, Korea) was performed. The waist ratio was calculated as follows: [waist diameter measured at the midpoint from the first lumbar to top edge of the iliac crest/distance from the first lumbar to top edge of the iliac crest].

### Blood test: hematology and serum biochemistry

Blood was withdrawn from the auricular artery of all rabbits and collected in EDTA‐K2 tubes (BD, East Rutherford, NJ, USA) containing an anticoagulant at the prediet stage (week 0) and then at 3‐week intervals after commencing the HFD. RBC, hemoglobin, and platelets were measured using a hematology analyzer (ADVIA2120i; Siemens, Munich, Germany). Plasma was separated by centrifugation at 2000 ***g*** for 10 min at 4 °C. Total cholesterol (TCHOL), low‐density lipoprotein (LDL), high‐density lipoprotein (HDL), phospholipid, blood urea nitrogen (BUN), and creatinine concentrations, as well as aspartate aminotransferase (AST) and alanine aminotransferase (ALT) activities, were measured using an automated biochemistry analyzer (TBA 120FR, Toshiba, Tokyo, Japan).

### Echocardiography

Cardiac function was tested at the prediet stage (week 0) and then at 3‐week intervals after starting the HFD until week 9 using the Vevo 2100 ultrasound system with an MS200 transducer (VisualSonics, Toronto, ON, Canada). The rabbits were placed in a right lateral recumbency position under 1.5% isoflurane anesthesia and left ventricular contractility was measured in triplicate. The interventricular septum, left ventricular internal dimension, and left ventricular free wall thickness in diastole and systole were measured using two‐dimensional guided M‐mode tracings from the right parasternal short‐axis view just below the mitral valve at the level of the papillary muscle to calculate the ejection fraction (EF) and fractional shortening (FS).

### Necropsy and histopathology

All rabbits were euthanized by intravenously injecting pentobarbitone sodium (Lethabarb, Virbac Australia, Milperra, NSW, Australia). The liver was dissected, and the relative organ weight was determined. For histopathological analysis, the liver, pancreas, aorta, kidney, and adrenal gland were fixed in 10% neutral buffered formalin (BBC Biochemical, Mount Vernon, WA, USA) and embedded in paraffin wax. Tissue sections were cut to a thickness of 4 μm, and hematoxylin and eosin (H&E) staining and Masson's trichrome staining were performed using an automated staining machine (DAKO Coverstainer, WA, USA).

### Statistical analysis

All data are expressed as mean ± standard error of the mean (SEM). Statistical analyses were performed with multiple *t*‐tests using graphpad prism 8 (GraphPad Software Inc., San Diego, CA, USA). Statistical significance was set at *P* < 0.05.

## Results

### HFD increased weight gain and waist ratio

**Fig. 1:**
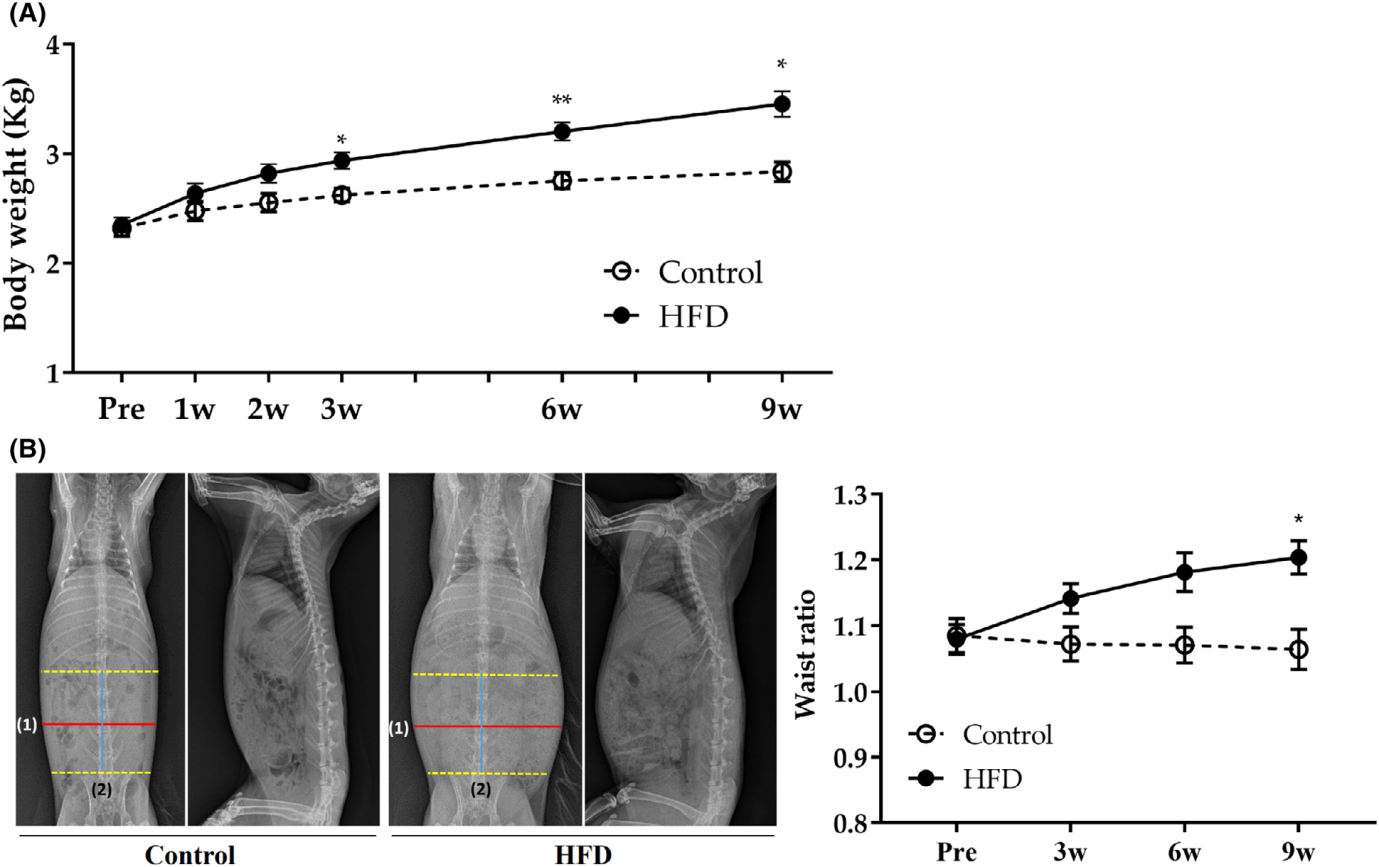
Change of body weight and Waist ratio. Changes in (A) body weight and (B) waist ratio. The representative picture of radiography at 9 weeks. The waist ratio was calculated as follows: [(1) waist diameter measured at midpoint from the first lumbar to top edge of the iliac crest/(2) distance from the first lumbar to top edge of the iliac crest]. Control group (*n* = 11) [3 weeks (*n* = 3), 6 weeks (*n* = 4), and 9 weeks (*n* = 4)]. HFD group (*n* = 22) [3 weeks (*n* = 6), 6 weeks (*n* = 8), and 9 weeks (*n* = 7)]. Statistical analyses were performed with multiple *t*‐tests. Data are expressed as mean ± SEM. **P < 0.01 or *P < 0.05 versus the control group.

The body weights of both groups gradually increased until the third week of the diet treatment, with no significant differences between the control and HFD groups. However, the body weight of the HFD group continued to increase after 6 weeks and was significantly higher than that of the control group, whereas the body weight of the control group reached a plateau and remained constant until the end of the experiment (Fig. 1A). The waist ratio of the HFD group continued to increase in contrast to the control group and significantly higher than that of the control group at 9 weeks (Fig. 1B).

### HFD significantly increased serum lipid profiles

**Fig. 2:**
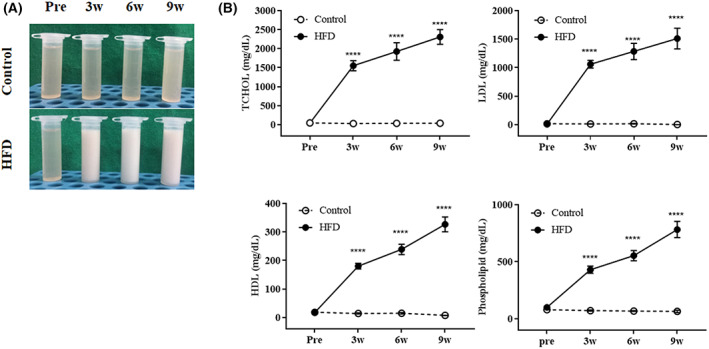
Change of lipid profile. Changes in (A) Gross inspection of serum in all groups at 0 (Pre), 3, 6, and 9 weeks in rabbits. (B) Changes in lipid profiles. Changes in the TCHOL, LDL, HDL, and phospholipid levels at 0 (Pre), 3, 6, and 9 weeks in rabbits. Control group (*n* = 11) [3 weeks (*n* = 3), 6 weeks (*n* = 4), and 9 weeks (*n* = 4)]. HFD group (*n* = 22) [3 weeks (*n* = 6), 6 weeks (*n* = 8), and 9 weeks (*n* = 7)]. Statistical analyses were performed with multiple *t*‐tests. Data are expressed as mean ± SEM. ****P < 0.0001 versus the control group.

The plasma of rabbits in the HFD group became cloudy and milky after 3 weeks, in contrast to those in the control group, which remained clear throughout the experiment (Fig. 2A). The TCHOL, LDL, HDL, and phospholipid concentrations in the plasma of HFD rabbits were significantly higher than those of the control group after 3 weeks and by 58‐, 388‐, 42‐, and 12‐fold at 9 weeks, respectively (Fig. 2B). These findings suggest that aberrant lipid metabolism is related to diet‐induced changes in the liver. Therefore, we next evaluated the time‐course changes in liver enzyme levels and hepatic pathology.

### HFD‐induced hepatomegaly, alterations in liver enzymes activities, and steatosis

**Fig. 3:**
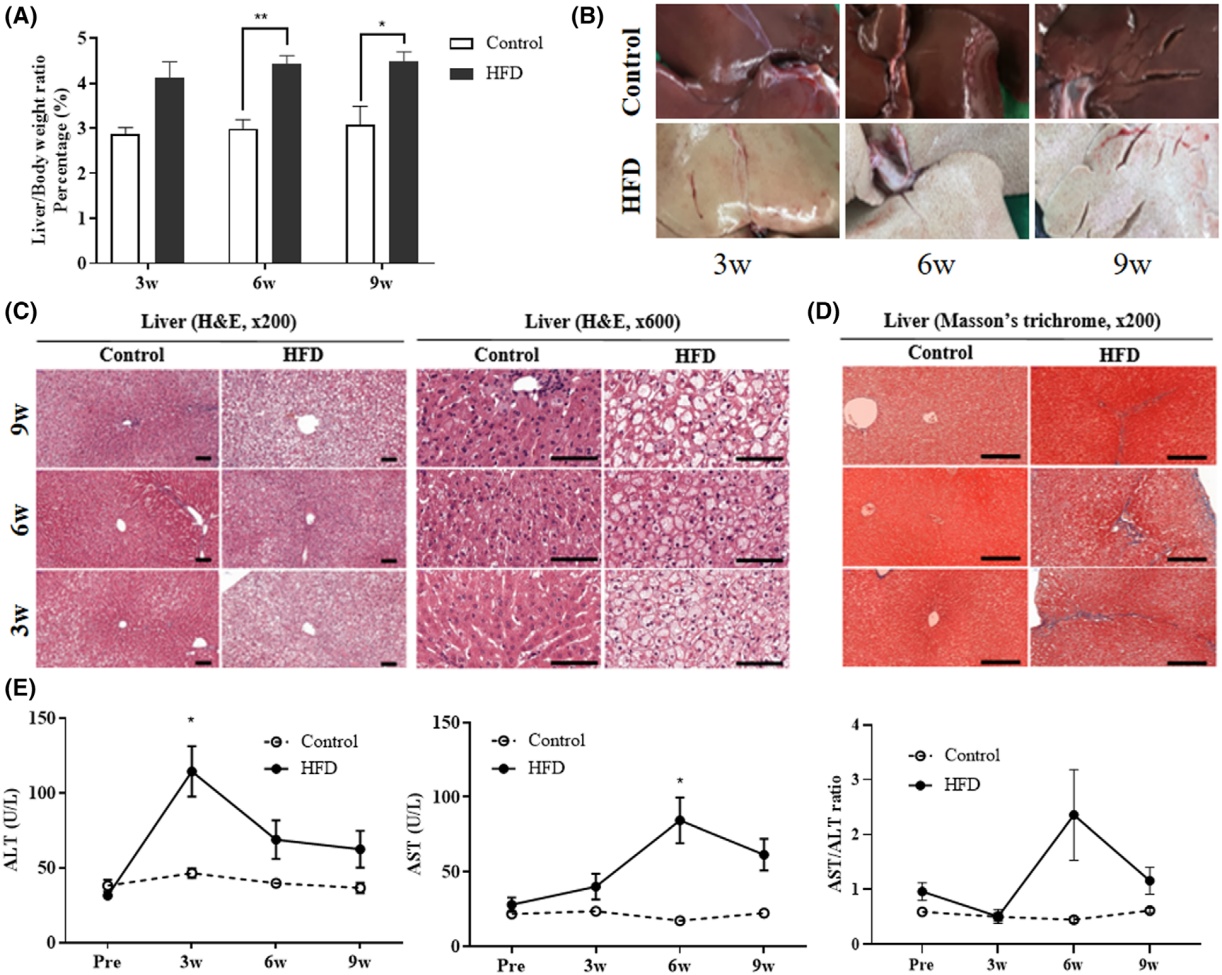
Pathological and morphological changes in the liver. (A) Ratios of liver/body weight at 0 (Pre), 3, 6, and 9 weeks in rabbits. (B) Gross appearance of liver, (C) Histopathological changes in the liver. H&E staining of liver in rabbits fed HFD [original magnifications were ×200 and ×600]. Liver from rabbits in the control group and those fed HFD for 3, 6, and 9 weeks. Lipid accumulation (microvesicular steatosis predominantly), hepatocyte hypertrophy, and loss of lobular structure were observed in the liver of rabbits fed the HFD for 3, 6, and 9 weeks compared with the control. Scale bars = 100 μm. (D) Masson's trichome staining of liver from rabbits fed HFD [original magnification was ×200]. Liver from rabbits in the control and those fed HFD for 3, 6, and 9 weeks. Fibrogenesis (blue) was observed in the liver of rabbits fed the HFD for 3, 6, and 9 weeks compared with control. Scale bars = 250 μm. (E) ALT, AST, and AST/ALT levels in the blood at 0, 3, 6, and 9 weeks in rabbits. Statistical analyses were performed with multiple *t*‐tests. Control group (*n* = 11) [3 weeks (*n* = 3), 6 weeks (*n* = 4), and 9 weeks (*n* = 4)]. HFD group (*n* = 22) [3 weeks (*n* = 6), 6 weeks (*n* = 8), and 9 weeks (*n* = 7)]. Statistical analyses were performed with multiple *t*‐tests. Data are expressed as mean ± SEM. **P < 0.01 or *P < 0.05 versus the control group.

The relative weight of the liver to the body was significantly higher in the HFD group than in the control group at 6 and 9 weeks. The liver/body weight ratio was 1.49‐ and 1.46‐fold higher on average in the HFD group at 6 and 9 weeks compared with the control group, respectively (Fig. 3A). The livers of rabbits fed HFD exhibited features of fatty liver with pale color and blunted edges, contrary to the normal features of the control groups (Fig. 3B). Only the HFD group developed Lipid accumulation (microvesicular steatosis predominantly), hepatocyte hypertrophy, loss of lobular structure, and fibrosis at 3, 6, and 9 weeks (Fig. 3C,D). ALT and AST activities and AST/ALT of the HFD group were significantly elevated than those of the control group at 3, 6, and 9 weeks (Fig. 3E).

### HFD significantly decreased RBC and hemoglobin concentrations and increased platelet concentration

**Fig. 4:**
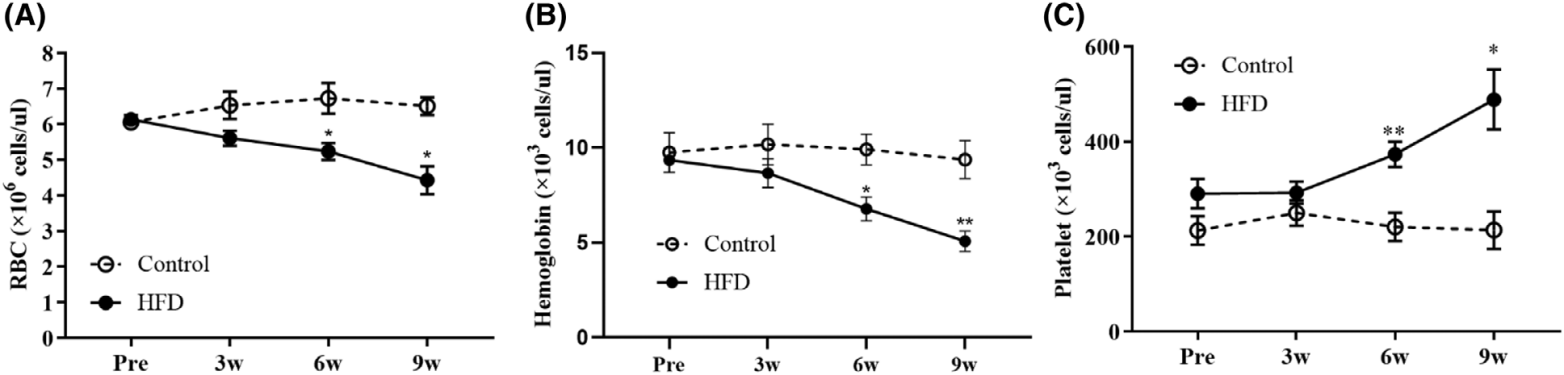
Changes in (A) RBC, (B) hemoglobin, and (C) platelet concentrations in the blood at 0 (Pre), 3, 6, and 9 weeks in rabbits. Control group (*n* = 11) [3 weeks (*n* = 3), 6 weeks (*n* = 4), and 9 weeks (*n* = 4)]. HFD group (*n* = 22) [3 weeks (*n* = 6), 6 weeks (*n* = 8), and 9 weeks (*n* = 7)]. Statistical analyses were performed with multiple *t*‐tests. Data are expressed as mean ± SEM. **P < 0.01, or *P < 0.05 versus the control group.

The HFD induced significant differences in the RBC, hemoglobin, and platelet concentrations in the blood compared with the control group. After 3 weeks, RBC and hemoglobin concentrations in the plasma of the HFD group were significantly lower than those of the control group and gradually decreased in contrast to the control group (Fig. 4A,B). The platelet concentration in the plasma was significantly higher in the HFD group than in the control group after 6 weeks (Fig. 4C).

### HFD‐induced histopathological and functional changes in the cardiovascular system

**Fig. 5:**
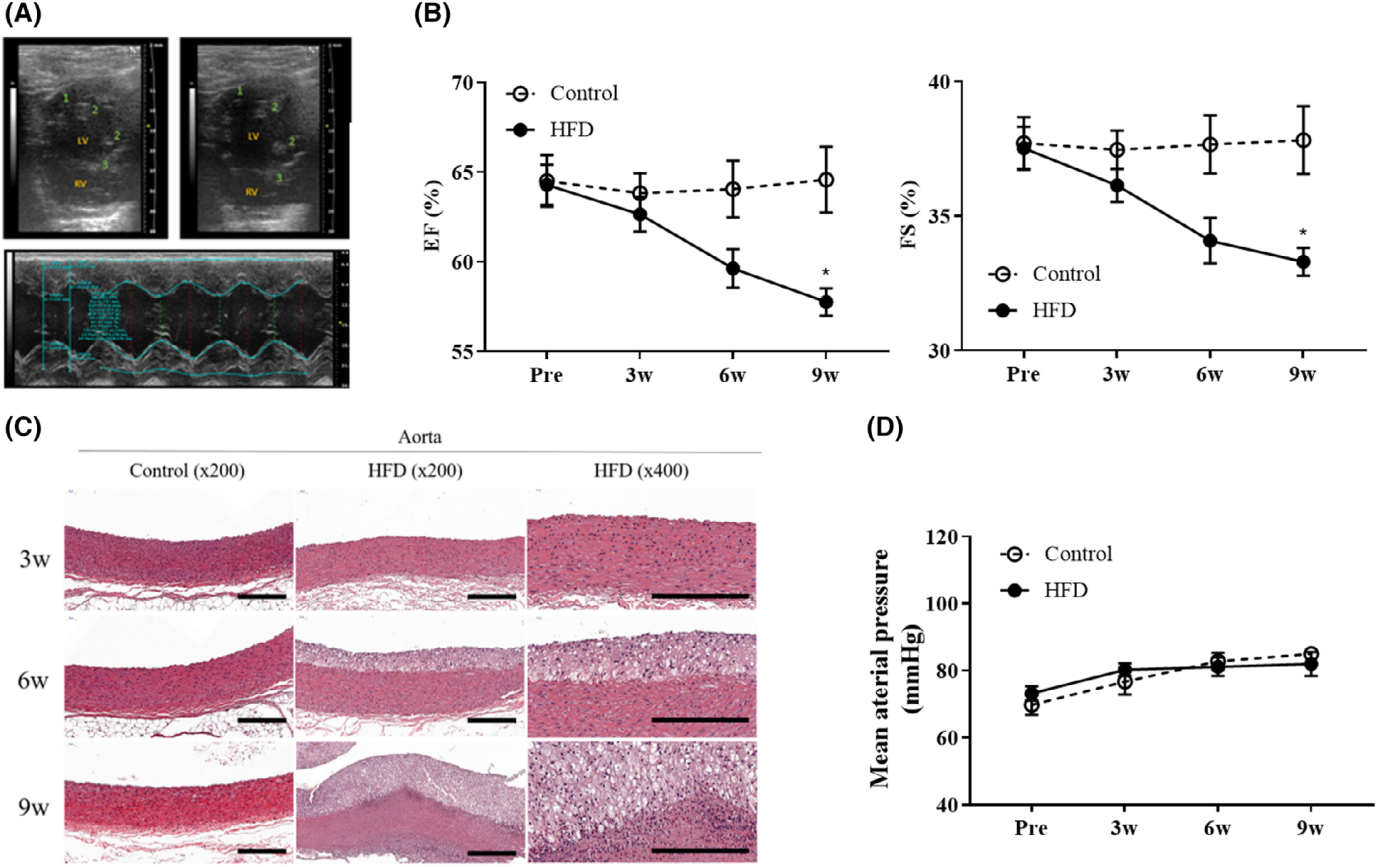
Changes in the cardiovascular system. (A) Echocardiography of the left parasternal short‐axis view (upper) with two‐dimensional guided M‐mode tracing (lower) at the level of the papillary muscle in the left ventricle. (B) Index of cardiac contractility. EF% and FS%. (C) Transverse section of the H&E‐stained aorta of the control and HFD‐fed rabbits at 3, 6, and 9 weeks. In the 6 and 9 weeks HFD groups, atherosclerotic lesions represent proliferated collagen fibers, lipid accumulation (6, 9 weeks), and infiltrated inflammatory cells (9 weeks). Scale bars = 250 μm. (D) Mean arterial pressure (MAP) measured in the auricular artery. Values are means of three measurements taken within 3 min in all rabbits. Statistical analyses were performed with multiple *t*‐tests. Control group (*n* = 11) [3 weeks (*n* = 3), 6 weeks (*n* = 4), and 9 weeks (*n* = 4)]. HFD group (*n* = 22) [3 weeks (*n* = 6), 6 weeks (*n* = 8), and 9 weeks (*n* = 7)]. Data are expressed as mean ± SEM. *P < 0.05 versus the control group.

Ejection fraction (EF)% and fractional shortening (FS)%, indicating the function of cardiac contractility, gradually decreased in the HFD group compared with those in the control group. EF% and FS% of the HFD group were significantly lower than those of the control group at 9 weeks (Fig. 5A,B). Atherosclerotic plaques were found in the aorta at 3, 6, and 9 weeks, with inflammatory cell infiltration at 9 weeks in the HFD group, contrary to the normal aorta features in the control group, while there were no pathological lesions in the carotid artery in either group (Fig. 5C). The mean arterial pressure (MAP) in both groups increased, and there was no significant difference between the groups (Fig. 5D).

### HFD‐induced histopathological changes in the lipid accumulation in the pancreas and adrenal gland and vacuolation in the pancreas

**Fig. 6:**
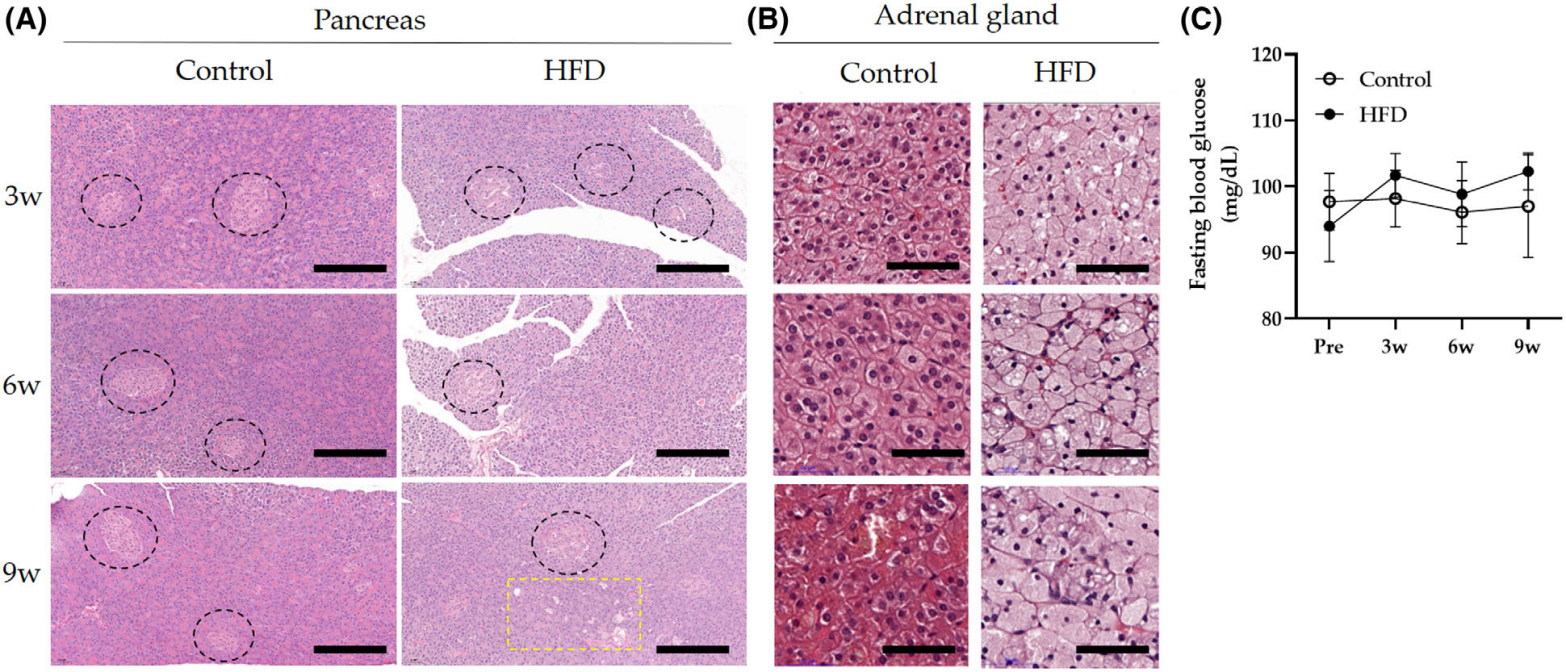
H&E staining of the pancreas and zona fasciculata of the adrenal gland and fasting blood glucose concentration in rabbits. (A) Negative control and HFD groups at 3, 6, and 9 weeks. In HFD groups, β‐islet represented vacuolization (3, 6, and 9 weeks) and lipid accumulation (9 weeks) in the pancreas. Scale bars = 200 μm. Black circle = β‐islet, yellow quadrangle = lipid accumulation. (B) Lipid accumulation, hypertrophy, and loss of lobular structure were observed in the adrenal gland of HFD‐fed rabbits. Scale bars = 50 μm. (C) Changes in fasting blood glucose concentrations at 0 (Pre), 3, 6, and 9 weeks in rabbits. Data are expressed as mean ± SEM.

Lipid accumulation was observed in the pancreas, adrenal gland, and liver of the HFD group at 3, 6, and 9 weeks. Significant pathological changes such as hypertrophy, inflammatory cell infiltration, fibrosis, and hemorrhage in the adrenal gland were also observed. β‐Islet vacuolation was found in the pancreas of the HFD group at 3, 6, and 9 weeks. There was no statistical difference in fasting blood glucose between the two groups (Fig. 6).

### HFD‐induced clinical and histopathological changes in the kidney

**Fig. 7:**
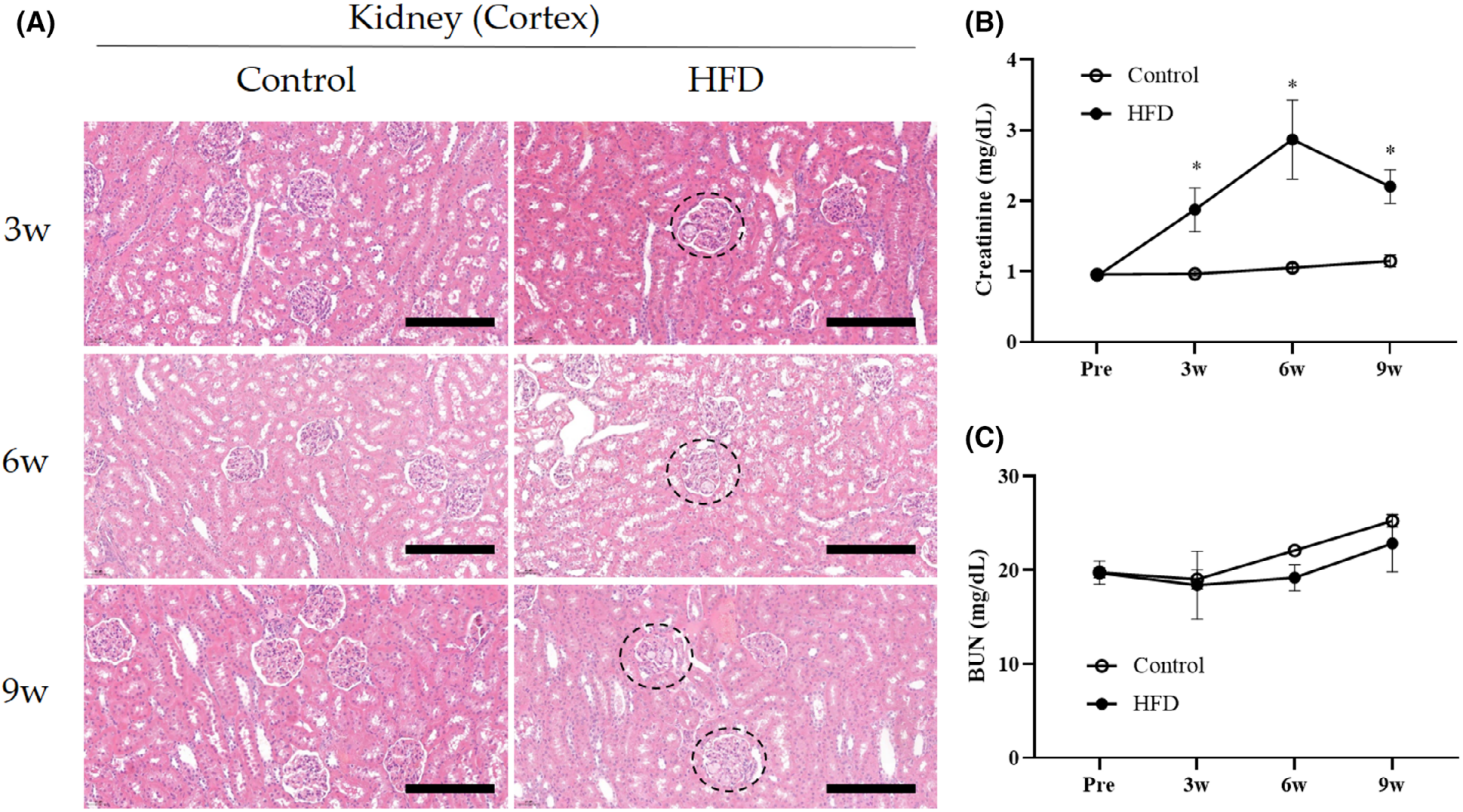
H&E staining of the kidney (cortex), creatinine, and BUN concentration in rabbits. H&E staining of the kidney cortex of rabbits in the negative control and HFD‐fed groups at 3, 6, and 9 weeks. (A) In the HFD groups, the glomerulus represents glomerulosclerosis (3, 6, and 9 weeks). Scale bars = 200 μm. Black circle = glomerulosclerosis. (B) Changes in creatinine concentrations at 0 (Pre), 3, 6, and 9 weeks in rabbits. (C) Changes in BUN concentrations at 0 (Pre), 3, 6, and 9 weeks in rabbits. Control group (*n* = 11) [3 weeks (*n* = 3), 6 weeks (*n* = 4), and 9 weeks (*n* = 4)]. HFD group (*n* = 22) [3 weeks (*n* = 6), 6 weeks (*n* = 8), and 9 weeks (*n* = 7)]. Statistical analyses were performed with multiple *t*‐tests. *P < 0.05 versus the control group.

Creatinine concentration in the serum of the HFD group was significantly higher than that of the control group at 3, 6, and 9 weeks. The blood urea nitrogen (BUN) concentration between HFD and control groups was not significantly different. Glomerulosclerosis was observed in the kidneys of the HFD group at 3, 6, and 9 weeks (Fig. 7).

## Discussion

Obesity, which is attributed to the consumption of an HFD, is a risk factor associated with fatty liver diseases, diabetes, and atherosclerosis in industrialized societies, with a dramatically increasing prevalence in the United States, Asia, and Europe [27, 28, 29]. Diabetes can be induced by DNA methylation, thereby reducing the transcriptional activity of beta cell genes and inducing insulin resistance after the consumption of an HFD. HFD‐associated obesity primarily causes nonalcoholic fatty liver disease (NAFLD) owing to endoplasmic stress, perturbation of autophagy, mitochondrial dysfunction, and inflammatory responses, causing abdominal pain, nausea, jaundice, and edema [30]. In addition, HFD induces pro‐inflammatory conditions that develop atherosclerosis with aberrant inflammatory responses in the arterial walls. Atherosclerosis is responsible for myocardial infarction, cerebral infarction, and stroke, leading to temporary vision loss, difficulty speaking, and kidney failure [31]. Pre‐emptive efforts are required to reduce HFD exposure because of the interrelated networks of metabolic syndromes in other diseases [1, 10, 11]. In this study, we characterized multiple time‐dependent HFD‐induced pathological, cardiovascular, and morphological changes to provide basic data showing the progression from an acute response to adaptation and aid the development of novel drugs using a hyperlipidemia rabbit model.

We observed that HFD significantly increased the body weight and waist ratio compared with the body weight plateau of the control after 3 and 9 weeks, respectively. The energy density of fat (9 kcal·g^−1^) was more than twice that of carbohydrate or protein (4 kcal·g^−1^) [32], validating the relatively high‐fat composition in the body weight of HFD‐fed rabbits. The finding that HFD increased body weight or waist ratio is consistent with many previous findings [1, 4, 23, 25, 26].

High‐fat diets remarkably altered the lipid profile compared with the results of previous studies [6, 26, 33]. TCHOL, LDL, HDL, and phospholipids were significantly higher in the HFD group than in the control group at 3 weeks and continued to increase until 9 weeks. Lipid dysregulation is a risk factor for the cardiovascular system, liver, pancreas, and kidney [3, 34, 35, 36]. Recent reports observed that HFD first alters the lipid profile, followed by other factors such as histopathology. Prim *et al*. [37] reported a high lipid profile, with no histopathological changes in the aorta 56 days after HFD consumption in rabbits.

Lipid dyslipidemia and steatosis of the liver appeared at 3 weeks, whereas atherosclerotic plaques with proliferated collagen fiber and lipid accumulation, and inflammatory cell infiltration in the aorta were observed at 6 and 9 weeks after HFD consumption, respectively. These results suggest that feeding an HFD for a longer period is recommended for histopathological research related to the cardiovascular system.

Hypercholesterolemia is the most evident cause of cardiovascular diseases [38]. Cholesterol is a required component of the animal cell membrane, where it maintains the barrier function to the environment and modulates fluidity and signaling molecules. Cholesterol is also the sole precursor of steroid hormones and plays a major role in myelin sheath formation surrounding axons [39]. In the bloodstream of humans and other vertebrates, cholesterol is transported as lipoprotein particles, which are divided into two major proteins: LDL and HDL [40]. The normal physiological mechanisms of LDL are associated with its ability to transport cholesterol to cells when the cell requires more cholesterol [41]. However, the relationship between high levels of plasma LDL‐cholesterol and the risk of cardiovascular disease is well‐established. Oxidized LDL particles result in endothelial dysfunction, leading to the expression of adhesion molecules with monocyte recruitment from the subendothelial space, inducing atherosclerosis [42, 43]. LDL levels are also associated with diabetes, myocardial infarction, and hypertension. By contrast, there has been ongoing controversy surrounding the benefits and risks of HDL [44]. HDL is an essential component that mediates cellular cholesterol efflux, delivers cholesterol from extrahepatic tissues to the liver, and serves as a preferential precursor for bile acid biosynthesis with other beneficial mechanisms, including anti‐oxidation, anti‐thrombosis, and anti‐inflammation [45]. Blood HDL levels are considered strong inverse predictors of future cardiovascular morbidity and mortality because HDLs have multiple anti‐atherogenic effects by taking up cholesterol from foam cells localized to lipid accumulation on blood vessel walls, inhibiting LDL oxidation, and limiting the inflammatory process underlying atherosclerosis [44, 46, 47, 48]. However, despite the beneficial effect of HDL on the body, high HDF concentration may adversely affect the cardiovascular system.

Low or extremely high HDL levels are associated with all‐cause mortality, including cardiovascular disease, while slightly high HDL level suppresses mortality [49]. Atherosclerotic disease is responsible for the increased risk of mortality associated with very high HDL levels. HDL may be functionally compromised owing to an extremely high HDL level, inducing normal HDLs to be detrimental [50], which may be associated with a higher risk of atherosclerosis [51]. Endothelial function impairment has been observed in patients with extremely high and low HDL concentrations [52]. Although HDL has anti‐inflammatory benefits in the absence of inflammation, when inflammation arises, HDL exacerbates inflammation, such as atherosclerosis [53]. The European Society of Cardiology/European Atherosclerosis Society (ESC/EAS) dyslipidemia guidelines reported that the risk of atherosclerosis is elevated when the HDL concentration exceeds 90 mg·dL^−1^. Our data showed that HFD substantially increased TCHOL, LDL, HDL, and phospholipid levels, by 58‐, 388‐, 42‐, and 12‐fold, respectively, compared with the control group at week 9. Phospholipids and cholesterols, one of the major structure of cell membranes, should exist in a constant ratio in the cell membrane such as erythrocytes. However, Hypercholesterolemia can induce the irreversible change in cell membrane due to excessive cholesterol proportion in cell membrane. It can consequently induce a morphological change in red blood cell aggregability and atherosclerosis [54, 55]. Our experimental results, in which the cholesterol elevation rate is much higher than the phospholipid elevation rate in serum, predict the occurrence of atherosclerosis. Therefore, we can predict the adverse effects of severe hyperlipidemia, including extremely high concentrations of LDL and HDL, in the body.

Consuming excess fat is toxic to the liver because of the harmful fat accumulation, causing damage and building scar tissue in the liver [56]. In this study, HFD resulted in blunted, obtuse, and rounded lobe tips, indicating hepatomegaly and hepatic steatosis with the pale color of the liver and alteration of liver enzyme activities. ALT and AST activities were significantly higher in the HFD group than in the control group at 3 and 6 weeks, respectively. Surprisingly, the AST/ALT ratio in the HFD group was significantly higher than that in the control group. This biochemical change is a diagnostic marker for alcoholic fatty liver disease in the medical population [57]. As fibrosis progresses in the liver, ALT activity commonly decreases and AST to ALT gradually increases due to ALT production decrease [58]. We observed fibrosis in the liver at 3, 6, and 9 weeks. In addition, the half‐life of ALT in rabbits is about 5 h, which is relatively very short compared with other species, about 50 h for dogs or humans, or about 25 h for mice [59]. Therefore, the transient increase of ALT at 3 weeks was due to liver fibrosis and its short life.

Steatosis was observed within 3 weeks, with microvacuoles detected in hepatocytes. The presence of microvacuoles in HFD‐induced steatosis in rabbits indicates reactive oxygen species (ROS) stress, which can affect lipid metabolism in the mitochondria [60, 61]. An HFD can induce liver injury and insulin resistance by oxidative stress, and increased ROS level may cause lipid peroxidation and subsequent inflammatory response, leading to fibrogenesis, followed by activation of stellate cells [62].

We observed comprehensive changes in the entire body system induced by high lipid profile levels. First, the hematologic evaluation showed a decrease in RBC and hemoglobin concentrations, an increase in platelet concentration over 9 weeks, and an increase in white blood cells (WBCs), monocytes, and lymphocytes up to 6 weeks in rabbits fed HFD. These results are highly similar to those of previous studies [23, 63]. Studies have reported that an HFD induces the production of oxygen species, increasing the auto‐oxidation rate of hemoglobin, which promotes the conversion of HbO_2_ to methemoglobin. This conversion leads to a decrease in RBC and hemoglobin concentrations, and oxidative stress can cause hemolytic anemia in a high‐cholesterol diet [64]. The increase in platelets in the blood after HFD consumption can be affected by atherosclerosis pathogenesis [63, 65]. Changes in the microenvironment, such as hyperlipidemia, can induce endothelial damage, increasing adhesion molecules, such as intercellular adhesion molecule‐1, vascular cell adhesion molecule‐1, and P‐selectin, and adhesion proteins, such as von Willebrand factor (vWF) and fibrin, thereby activating the adhesion of platelets to the damaged site [65].

Furthermore, we observed functional and pathological changes in the cardiovascular system. Atherosclerotic plaques and inflammatory cell infiltration in the aorta can decrease blood flow velocity [43]. Therefore, we observed dynamic blood flow changes using echocardiography to calculate EF% and FS%. Echocardiography is a noninvasive method for measuring cardiac function with gentle restraint or light anesthesia, which does not affect cardiac function [66]. EF and FS are commonly used parameters to evaluate contractility as they are related to the end‐diastolic volume and are influenced by changes in afterload. Because cardiac output is the main parameter determining total oxygen delivery to the tissues, FS and EF values can indicate alterations in the blood delivery system in the body [67]. In the present study, we demonstrated that HFD‐induced atherosclerosis resulted in decreased cardiac contractility, consistent with the findings of many previous studies [2, 68].

A decrease in EF of 50% or more is required to be diagnosed as cardiac dysfunction [69]. However, the reduction of about 12% in this study is relatively small. In addition, it is difficult to define heart failure because there were no clinical symptoms such as dyspnea, fatigue, and edema due to cardiac dysfunction. However, the EF and FS values of the HFD group continued to decrease on average during the experiment period, and in the 9th week, the last week of the experiment, they decreased significantly compared with the control group. Therefore, the EF value may be reduced to less than 50%, which can be diagnosed as heart abnormality with long‐term HFD. Also, there was no difference in blood pressure between the two groups in this study. EF reduction at week 9 and confirmation of atherosclerosis may predict further EF reduction and hypertension in the future.

Creatinine is a nonprotein nitrogenous substance produced by muscle metabolism in which creatine in the muscle is converted into creatinine. Urea is the primary serum metabolite derived from the breakdown of dietary protein by the liver, and the BUN level indicates the concentration of urea nitrogen in the blood [70]. Our data showed that creatinine levels significantly increased with glomerulosclerosis and lipid accumulation in the kidney in the HFD group, consistent with multiple previous studies [71, 72, 73]. Glomerulosclerosis is a histologic lesion in which scar tissue and thickness of the glomerular basement membrane develop on the glomeruli, causing them to lose function. The pathological lesion might increase the creatinine level in the blood through anatomic‐physiologic dysfunction of creatinine excretion from the renal tubule. No difference was observed in the BUN levels in both groups. Although creatinine and BUN levels are key factors in determining renal function, creatinine level is a more important factor than BUN level because BUN is affected by extrarenal factors [74]. BUN may increase gastrointestinal bleeding, dehydration, and catabolic states and decrease severe liver failure by impaired conversion of ammonia to urea in the liver [75]. Therefore, creatinine is considered a specific indicator of renal glomerular filtration rate [74].

HFD aggravates pancreatic injury in animal models because dietary fat is a highly stimulating factor for pancreatic secretion. A previous study demonstrated that an HFD induces hyperlipidemia with changes in pancreatic endocrine and exocrine functions. In addition, relationships between HFD‐induced hyperlipidemia and oxidative stress, followed by microcirculation, are well‐established.

Our study revealed that HFD induced substantial cytomorphologic changes in the pancreas, although the glucose levels did not differ between HFD and control groups. Pancreatic β‐cell islet vacuolation was observed in the HFD group at 3, 6, and 9 weeks through β‐cell autophagy, which integrates processes in the regulation of cell growth, development, and homeostasis, where it helps to maintain a balance between biogenesis and cellular component degradation [76]. HFD can stimulate the autophagy of β‐cells, whereas β‐cell dysfunction induced glucose intolerance in autophagy‐deficient mutant animals [76]. Therefore, we determined that the cytomorphological changes in β‐cell islet vacuolation in the pancreas with normal glucose levels in our study were normal responses to protect the body. However, lipid accumulation in the pancreas was observed in the HFD group at 9 weeks, suggesting that lipotoxicity should be considered in the future. Long‐term HFD exceeding the normal pancreatic autophagy capacity causes oxidative stress and microcirculatory blood flow disturbances in the pancreas, inducing fat accumulation with lipotoxicity and a cascade of pancreatic dysfunction, such as chronic nonalcoholic fatty pancreas disease [62].

Our study results revealed the adverse effects of HFD and demonstrated time‐dependent changes in many organs to predict disease development. However, the long‐term effects of HFD could not be predicted because more than 9 weeks of HFD could not be confirmed. Therefore, we need further study to confirm the changes in the whole body system by exposure to long‐term HFD.

## Conclusions

Overall, our comprehensive investigation provides useful global reference data on the time‐dependent effects of HFD on the body. First, we observed hyperlipidemia, steatosis, and fibrogenesis with ALT activity elevation, β‐islet vacuolization, lipid accumulation and hypertrophy in the adrenal gland, and glomerulosclerosis and creatinine level elevation in the kidney at 3 weeks. Second, we recorded decreases in RBC and hemoglobin levels with increased platelet levels, atherosclerosis, hepatomegaly, and AST activity elevation at 6 weeks. Third, we detected a decrease in the contractility of the heart and lipid accumulation in the pancreas at 9 weeks. This is the first observation of lots of factors including blood parameter; including lipid and liver function enzyme, cardiodynamic, and pathological changes in multiple organs; including the liver, aorta, pancreas, adrenal gland, and kidney, over time after exposure to an HFD. These comprehensive results of multiple time‐dependent pathophysiological changes will contribute to the prediction of disease development by HFD and establish a rabbit model for the efficacy evaluation of new drugs associated with obesity.

## Conflict of interest

The authors declare no conflict of interest.

## Author contributions

WJ and TO conceived and designed the project. G‐HL wrote and edited the paper. G‐HL and HHY conducted animal studies and acquired data. K‐KK performed tissue slide preparation and pathology. G‐HL analyzed and interpreted the data.

## Data Availability

The data presented in this study are available upon request from the corresponding author.
